# Supplementary material for: Determination of Ligand Profiles for Pseudomonas aeruginosa Solute Binding Proteins
Source: Int J Mol Sci. 2019 Oct 17;20(20):5156. doi: 10.3390/ijms20205156 (PMC6829864; doi:10.3390/ijms20205156)
Supplement: Supplementary file 1 [file ijms-20-05156-s001.pdf]

Supplementary material

to

Determination of ligand profiles for *Pseudomonas aeruginosa* solute binding proteins

by

Matilde Fernández, Miriam Rico-Jiménez, Álvaro Ortega, Abdelali Daddaoua, Ana Isabel García García, David Martín-Mora, Noel Mesa Torres, Ana Tajuelo, Miguel A. Matilla and Tino Krell

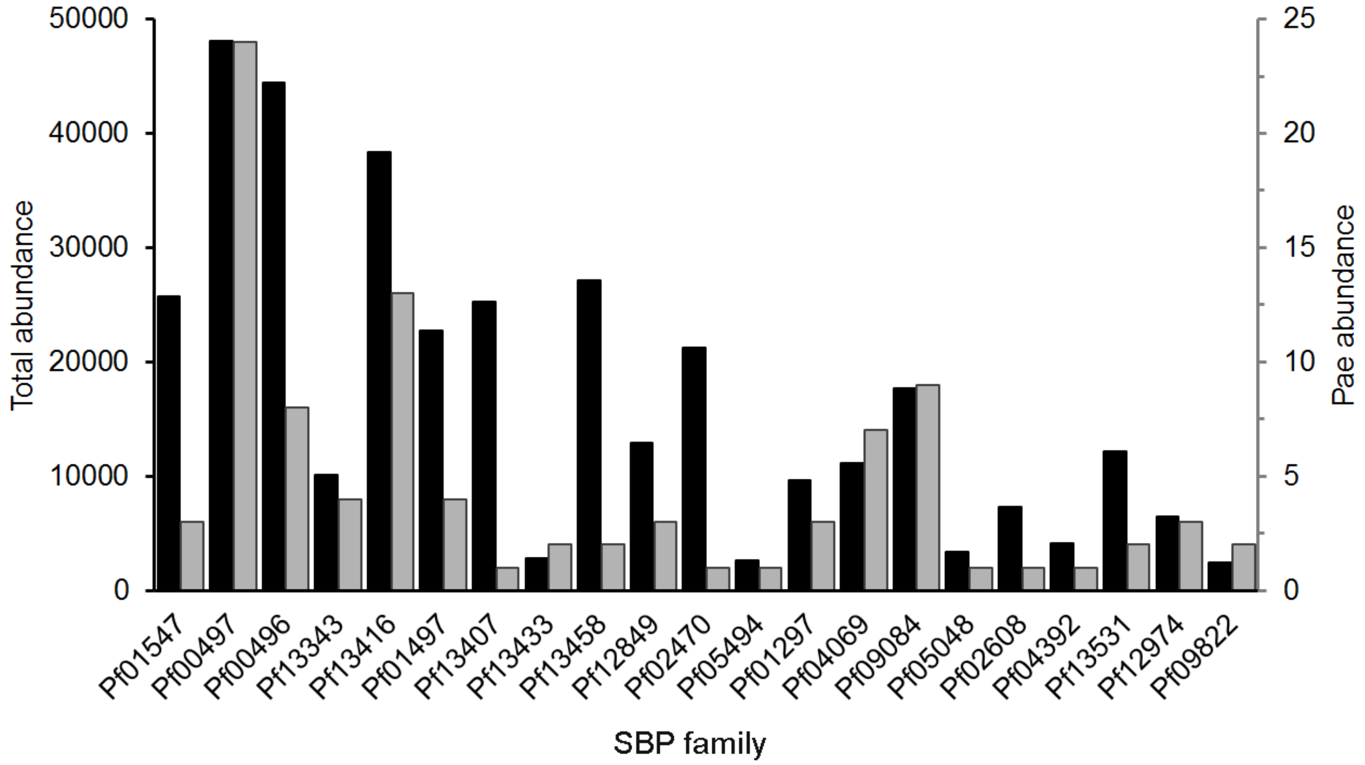

Supp. Fig. 1) The abundance of SBPs in the different Pfam protein families (black columns and left y-axis) and that in *P. aeruginosa* PAO1 (grey columns and right y-axis).

```
                  10        20        30        40        50        60
                   |         |         |         |         |         |
Atu4243      --------MRSKLVLSTCAALFALGAAAQANDLVFSSWGGTTQDAQKAAWAEKFMVETGI
PA0222       MFKSLHQYAHVFSRLSLFGLAFAAAAQAQSQSLTVISFGGATKAAQEQAYFKPFERSGGG

                  70        80        90       100       110       120
                   |         |         |         |         |         |
Atu4243      NVLQ-DGPTDYGKLKAMVEANGVTWDVVDVEGDYAAQAGPKGLLEKLDFS-VIDKTKLDP
PA0222       QVVAGEYNGEMAKVKAMVDVGKVSWDVVEVESPELLRGCDEGLFERLDPARFGDPAQFVP

                 130       140       150       160       170       180
                   |         |         |         |         |         |
Atu4243      RFVTDYSVGSFYYSFVIGCNVDSVSACPKSWADLFDTAKFPGKRTFYKWSAPGVIEAALL
PA0222       GTFSECGVATYVWSMVMAYDSTKLARAPQSWADFWNVREFPGKRGLRKG-AKYTLEVALL

                 190       200       210       220       230       240
                   |         |         |         |         |         |
Atu4243      ADGVTADKLY-----PLDLDRAFKKLDTIKSDIIWWSGGAQSQQLIASAEAPFGSVWNGR
PA0222       ADGVKAEDLYKVLATPEGVSRAFAKLDQLKPNIQWWEAGAQPPQWLAAGDVVMSAAYNGR

                 250       260       270       280       290       300
                   |         |         |         |         |         |
Atu4243      MTALEQSGVKVETSWAQNITAADSLVVPKGTKNKDAAMKFIALATSAQAQADMATATGYA
PA0222       IAAAQKEGVKLAIVWPGSLYDPEYWAVVKGTPNKALAEKFIAFASQPQTQKVFSEQIPYG

                 310       320       330       340       350
                   |         |         |         |         |
Atu4243      PVNIESAKLMDPKIAKSLPDQQT--ESQVNADMNYWAQHRDEIGERWYAWQAK
PA0222       PVHKGTLALLPKTVQEALPTAPANLEGARAVDAEFWVDHGEELEQRFNAWAAR
```

Supp. Fig. 2) Sequences alignment of the specific GABA binding proteins Atu4243 of *Agrobacterium fabrum* and PA0222 of *P. aeruginosa*. The alignment was carried out using the CLUSTALW multiple alignment tool of the NPSA suite [1]. The GONNET protein weight matrix was used in the slow pairwise alignment mode using a gap opening penalty of 10 and a gap extension penalty of 0.1. Red, identical; green, highly similar; blue, weakly similar.

Supp. Table 1) The repertoire of Solute Binding Proteins of *P. aeruginosa* PAO1. Information was extracted from the TransportDB [2] and Pfam [3], and manually curated. Shown in bold are proteins that have been analysed in this work.

Pfam/InterPro domain signature	SBP	Size (aa)	Annotation UniProt	TransportDB prediction
Pf01547: SBP_bac_1 Bacterial extracellular solute-binding protein	**PA1863 (ModA)**	**251**	**Molybdate-binding periplasmic protein ModA**	**molybdate**
	**PA2338**	**436**	**Probable binding protein of maltose/mannitol transporter**	**sugar**
	PA3190	420	Probable binding protein of ABC sugar transporter	sugar
Pf00497: SBP_bac_3 Bacterial extracellular solute-binding protein	PA0314	256	L-cysteine transporter of ABC system FliY	amino acid
	**PA0888 (AotJ)**	**259**	**Arginine/ornithine binding protein AotJ**	**amino acid**
	PA1260 (LhpP)	273	Amino acid ABC transporter periplasmic binding protein	amino acid
	**PA1342 (AatJ)**	**302**	**Probable binding protein component of ABC transporter**	**amino acid**
	PA1531	288	Uncharacterized protein	amino acid
	PA1604	301	Uncharacterized protein	-
	**PA2204**	**268**	**Probable binding protein component of ABC transporter**	**amino acid**
	PA2902	282	Uncharacterized protein	amino acid
	PA2923 (HisJ)	261	Periplasmic histidine-binding protein HisJ	histidine
	PA3261	248	Uncharacterized protein	amino acid
	PA3475 (PheC)	268	Cyclohexadienyl dehydratase	amino acid
	PA3858	341	Probable amino acid-binding protein	amino acid
	PA3865	263	Probable amino acid binding protein	amino acid
	PA3959	244	Uncharacterized protein	-
	PA4027	297	Uncharacterized protein	amino acid
	PA4049	248	Uncharacterized protein	amino acid
	PA4195	276	Probable binding protein component of ABC transporter	amino acid
	PA5076	266	Probable binding protein component of ABC transporter	amino acid
	PA5082 (DguC)	299	Probable binding protein component of ABC transporter	amino acid
	PA5137	251	Uncharacterized protein	amino acid
	PA5138	250	Uncharacterized protein	amino acid
	PA5139	248	Uncharacterized protein	amino acid

	PA5153	250	Lys, Arg, His, ornithine, octopine binding protein	amino acid
	PA5270	293	Uncharacterized protein	amino acid
Pf00496: SBP_bac_5 Bacterial extracellular solute-binding protein	PA1810 (NppA2)	615	Probable binding protein component of ABC transporter	oligopeptide
	PA1811 (NppA1)	609	Probable solute-binding protein	oligopeptide
	PA2058 (SppA)	602	Probable binding protein component of ABC transporter	oligopeptide
	PA4496 (DppA1)	537	Probable binding protein component of ABC transporter	dipeptide/oligopeptide
	**PA4497 (DppA2)**	**532**	**Probable binding protein component of ABC transporter**	**dipeptide/oligopeptide**
	**PA4500 (DppA3)**	**533**	**Probable binding protein component of ABC transporter**	**dipeptide/oligopeptide**
	PA4502 (DppA4)	531	Probable binding protein component of ABC transporter	dipeptide/oligopeptide
	PA5317 (DppA5)	526	Probable binding protein of ABC dipeptide transporter	dipeptide/oligopeptide
Pf13343: SBP_bac_6 Bacterial extracellular solute-binding protein	PA2377	424	Uncharacterized protein	iron(III)
	**PA4687 (HitA)**	**335**	**Ferric iron-binding periplasmic protein**	**iron(III)**
	PA5217	332	Probable binding protein component of ABC iron transporter	2-aminoethylphosphonate
	PA3250	352	Uncharacterized protein	2-aminoethylphosphonate
Pf13416: SBP_bac_8 Bacterial extracellular solute binding protein	PA0203	343	Probable binding protein component of ABC transporter	spermidine/putrescine
	**PA0222**	**352**	**Uncharacterized protein**	**spermidine/putrescine**
	PA0295	353	Probable periplasmic polyamine binding protein	spermidine/putrescine
	PA0300 (SpuD)	367	Putrescine-binding periplasmic protein SpuD	spermidine/putrescine
	PA0301 (SpuE)	365	Spermidine-binding periplasmic protein SpuE	spermidine/putrescine
	PA0323	347	Probable binding protein component of ABC transporter	spermidine/putrescine
	PA0602	344	Probable binding protein component of ABC transporter	spermidine/putrescine
	PA0604 (AgtB)	348	Probable binding protein component of ABC transporter	spermidine/putrescine
	PA1410	363	Probable periplasmic spermidine/putrescine-binding protein	spermidine/putrescine
	**PA2592**	**367**	**Probable periplasmic spermidine/putrescine-binding protein**	**spermidine/putrescine**
	PA2711	363	Probable periplasmic spermidine/putrescine-binding protein	spermidine/putrescine
	**PA3610 (PotD)**	**354**	**Polyamine transport protein PotD**	**spermidine/putrescine**
	PA4985	363	Uncharacterised protein	spermidine/putrescine
Pf01497: Peripla_BP_2	PA2913	323	Uncharacterized protein	cobalamin

Periplasmic binding protein	PA4045	265	Uncharacterized protein	iron-hydroxamate
	PA4159 (FepB)	301	Ferrienterobactin-binding periplasmic protein FepB	amino acid
	PA4708 (PhuT)	297	Heme-transport protein, PhuT	iron-hydroxamate
Pf13407: Peripla_BP_4 Periplasmic binding protein	**PA1946 (RspB)**	**319**	**Binding protein component of ABC ribose transporter**	**rhamnose**
Pf13433: Peripla_BP_5 Periplasmic binding protein	PA3364 (AmiC)	385	Aliphatic amidase expression-regulating protein	urea
	PA4858	421	Uncharacterized protein	urea
Pf13458: Peripla_BP_6 Periplasmic binding protein	**PA1074 (BraC)**	**373**	**Leu, Ile, Val, Thr and Ala binding protein**	**leucine/valine**
	**PA4913**	**374**	**Probable binding protein component of ABC transporter**	**leucine/valine**
Pf12849: PBP_like_2 PBP superfamily domain	PA0688 (LapA)	368	Alkaline phosphatase L	-
	PA0689 (LapB)	370	Uncharacterized protein	-
	PA5369 (PstS)	323	Phosphate-binding protein PstS	phosphate
Pf02470: MlaD protein	PA3213	312	Uncharacterized protein	toluene tolerance
Pf05494: MlaC protein	PA4453	215	Uncharacterized protein	-
Pf01297: ZnuA Zinc-uptake complex component A periplasmic	PA2407 (FpvC)	317	Probable adhesion protein	manganese/zinc ion
	PA2410 (FpvF)	305	Uncharacterized protein	manganese/zinc ion
	PA5498 (ZnuA)	307	Probable adhesin	zinc ion
Pf 04069: Substrate binding domain of ABC type glycine-betaine transport system	PA0030 (CosX)	307	Uncharacterized protein	choline
	PA3236 (BetX)	285	Probable glycine-betaine binding protein	glycine-betaine
	**PA3889 (OpuCC)**	**311**	**Probable binding protein component of ABC transporter**	**glycine-betaine**
	PA5096	322	Probable binding protein component of ABC transporter	glycine-betaine
	PA5103 (PuuR)	340	Uncharacterized protein	glycine-betaine
	PA5378 (CbcX)	312	Uncharacterized protein	choline
	PA5388 (CaiX)	312	Uncharacterized protein	choline
Pf09084: NMT1/THI5 like	PA0186	353	Probable binding protein component of ABC transporter	nitrate/sulfonate/taurine
	PA2594	319	Uncharacterized protein	nitrate/sulfonate/taurine
	PA2595	318	Uncharacterized protein	nitrate/sulfonate/taurine
	PA2596	328	Uncharacterized protein	nitrate/sulfonate/taurine
	PA2599	314	Uncharacterized protein	glycine-betaine
	PA3445	323	Uncharacterized protein	glycine-betaine
	PA3449	333	Uncharacterized protein	phosphonates
	PA3513	335	Uncharacterized protein	-
	PA3938	337	Probable periplasmic taurine-binding protein	glycine-betaine

Pf05048: Periplasmic copper-binding protein	PA3393 (NosD)	428	Probable ABC transporter binding protein NosD	-
Pf02608: ABC transporter substrate-binding protein	PA0146	365	Uncharacterized protein	sugar
Pf04392: ABC transporter substrate binding protein	PA3836	325	Uncharacterized protein	-
**Pf13531: Bacterial extracellular solute-binding protein**	**PA0283 (Sbp)**	**332**	**Sulphate-binding protein**	**sulphate/thiosulphate**
	**PA1493 (CysP)**	**332**	**Sulphate-binding protein of ABC transporter**	**sulphate/thiosulphate**
Pf12974: ABC transporter, phosphonate, periplasmic substrate-binding protein	PA3313	335	Uncharacterized protein	phosphonates
	PA3383	334	Binding protein component of ABC phosphonate transporter	phosphonates
	PA5101	265	Uncharacterized protein	phosphonates
Pf09822: ABC_transp_aux	PA3670	615	Uncharacterized protein	¿
	PA4039	600	Uncharacterized protein	¿
InterPro SSF53850: Periplasmic binding protein-like II superfamily	PA0777	250	Uncharacterized protein	amino acid
	PA5472	265	Uncharacterized protein	amino acid
InterPro IPR007487: ABC transporter, substrate-binding protein	PA1612	252	Uncharacterized protein	-

Supp. Table 2) Thermodynamic parameters derived from the microcalorimetric titration of different SBPs with ligands.

ORF (gene name)	Ligand	K_D (µM)	K_A (M^{-1})	ΔG (kcal/mol)	ΔH (kcal/mol)	ΔS (cal/mol/deg)	$T\Delta S$ (kcal/mol)
PA0222	GABA	0.29 ± 0.05	(3.44 ± 0.9) x10^6	-8.91 ± 0.16	-7.24 ± 0.62	5.6 ± 2.1	1.67 ± 0.62
PA0888 (*aotJ*)	L-Arg	0.12 ± 0.02	(8.22 ± 1) x10^6	-9.43 ± 0.07	-3.67 ± 0.05	19.3 ± 0.3	5.75 ± 0.09
PA1074 (*braC*)	L-Ala	0.21 ± 0.03	(4.76 ± 0.7) 10^6	-9.11 ± 0.09	-3.3 ± 0.1	19.5 ± 0.5	5.81 ± 0.15
	L-Ile	0.28 ± 0.05	(3.35 ± 0.4) 10^6	-8.90 ± 0.07	-6.25 ± 0.02	8.90 ± 0.2	2.65 ± 0.06
	L-Leu	0.29 ± 0.06	(3.32 ± 0.4) 10^6	-8.89 ± 0.07	-6.12 ± 0.70	9.3 ± 2.4	2.77 ± 0.70
	L-Val	0.07 ± 0.01	(1.35 ± 0.2) 10^7	-9.72 ± 0.09	-3.83 ± 0.10	19.8 ± 0.5	5.90 ± 0.15
	L-Thr	0.48 ± 0.1	(2.08 ± 0.4) x 10^6	-8.62 ± 0.11	-3.59 ± 0.97	16.9 ± 3.3	5.04 ± 0.98
	L-Homoserine	0.72 ± 0.1	(1.38 ± 0.2) 10^6	-8.37 ± 0.09	-2.69 ± 0.12	19.1 ± 0.5	5.69 ± 0.15
PA1342 (*aatJ*)	L-Asp	20.4 ± 5	(4.91 ± 1.0)10^4	-6.40 ± 0.12	0.85 ± 0.44	24.3 ± 1.5	7.24 ± 0.44
	L-Glu	1.4 ± 0.1	(4.70 ± 0.9) 10^5	-7.73 ± 0.11	-0.76 ± 0.02	23.4 ± 0.1	6.97 ± 0.3
PA1493 (*cysP*)	Thiosulphate	0.29 ± 0.2	(3.47 ± 0.2) 10^6	-8.92 ± 0.03	-11.82 ± 0.12	-9.7 ± 0.4	-2.89 ± 0.1
PA1863 (*modA*)	Chromate	0.44 ± 0.02	(2.25 ± 0.1) 10^6	-8.66 ± 0.03	-9.62 ± 0.06	-3.2 ± 0.2	-0.95 ± 0.06
	Molybdate	0.01 ± 0.001	(9.61 ± 1) 10^7	-10.9 ± 0.06	-7.66 ± 0.02	10.9 ± 0.2	3.25 ± 0.06
PA1946 (*rspB*)	D-Ribose	2.1 ± 0.1	(4.73 ± 0.1) 10^5	-7.74 ± 0.01	5.83 ± 0.04	45.5 ± 0.1	13.6 ± 0.03
	D-Allose	6.6 ± 0.1	(1.52 ± 0.05) 10^5	-7.07 ± 0.02	8.73 ± 0.10	53.0 ± 0.3	15.79 ± 0.1
PA2338	Mannitol	0.83 ± 0.2	(1.22 ± 0.3) 10^6	-8.30 ± 0.15	-9.07 ± 0.77	-2.6 ± 2.6	-0.77 ±0.77
PA2592	Putrescine	31 ± 4	(3.22 ± 0.3) 10^4	-6.15 ± 0.05	-2.7 ± 0.33	11.6 ± 1.1	3.46 ± 0.3
	Agmatine	15 ± 2	(6.55 ± 0.9) 10^4	-6.57 ± 0.08	-0.23 ± 0.03	21.3 ± 0.3	6.35 ± 0.1
PA3610 (*potD*)	Putrescine	4.8 ± 0.4	(2.06 ± 0.16) 10^5	-7.25 ± 0.05	-1.30 ± 0.03	20.0 ± 0.2	5.96 ± 0.06
	Cadaverine	65 ± 9	(1.54 ± 0.22) 10^4	-5.71 ± 0.08	0.55 ± 0.11	21.0 ± 0.5	6.26 ± 0.15
PA3889 (*opuCC*)	Glycine-betaine	3.0 ± 0.4	(2.56 ± 0.1) 10^5	-7.37 ± 0.05	-2.71 ± 0.30	15.6 ± 1.0	4.65 ± 0.3
	Ala-Ala	0.21 ± 0.02	(4.71 ± 0.3) 10^6	-9.10 ± 0.04	-23.8 ± 0.1	-49.3 ± 0.4	-14.7 ± 0.1
	Ala-Thr	0.98 ± 0.1	(1.09 ± 0.09) 10^6	-8.23 ± 0.05	-8.89 ± 0.11	-2.2 ± 0.4	-0.65 ± 0.1
	Ala-His	7.4 ± 0.4	(1.34 ± 0.7) 10^5	-6.99 ± 0.34	-6.68 ± 0.24	1.0 ± 1.4	0.30 ± 0.41
	Ala-Phe	6.1 ± 0.6	(1.62 ± 0.1) 10^5	-7.10 ± 0.04	-10.3 ± 0.65	-10.7 ± 2.2	-3.19 ± 0.65
	Gly-Val	6.3 ± 0.4	(1.57 ± 0.09) 10^5	-7.08 ± 0.03	-8.53 ± 0.33	-4.9 ± 1.1	-1.46 ± 0.32

Supp. Table 3) Thermal shift binding studies of PA4497 and PA4500 using compound arrays PM3B, PM6, PM7 and PM8. Listed are peptides that caused T_m increases superior to 3 °C.

well	ligand	T_m (°C)	ΔT_m (°C)
**PA4497**			
**PM8**			
G11	Ala-Ala-Ala	70.1	9.1
H1	Gly-Gly-Ala	68.6	7.6
H3	Gly-Gly-Gly	65.5	4.5
H4	Gly-Gly-Ile	66.1	5.1
H5	Gly-Gly-Leu	67.6	6.6
H6	Gly-Gly-Phe	69.4	8.4
H7	Val-Tyr-Val	66.0	5.0
H8	Gly-Phe-Phe	67.6	6.6
H9	Leu-Gly-Gly	64.2	3.2
H10	Leu-Leu-Leu	66.0	5.0
H11	Phe-Gly-Gly	64.7	3.7

**PA4500**			
**PM3B**			
H2	Ala-Gln	53.9	3.03
H4	Ala-Gly	58.9	7.96
H5	Ala-His	54.0	3.08
H6	Ala-Leu	54.7	3.76
H7	Ala-Thr	57.9	7.02
H12	Met-Ala	61.3	10.36
**PM6**			
A3	Ala-Ala	61.9	11.3
A4	Ala-Arg	57.4	6.9
A5	Ala-Asn	57.8	7.3
A7	Ala-Gly	58.1	7.5
A12	Ala-Pro	61.3	10.8
B1	Ala-Ser	57.7	7.1
B2	Ala-Thr	57.5	6.9
B5	Arg-Ala	55.6	5.1
G3	Ile-Ala	61.3	10.7
G4	Ile-Arg	57.4	6.9
G8	Ile-Ile	55.4	4.9
G10	Ile-Phe	54.0	3.5
G11	Ile-Pro	61.2	10.6
G12	Ile-Ser	57.8	7.3
H2	Ile-Tyr	54.9	4.4
H3	Ile-Val	61.0	10.4
H4	Leu-Ala	57.0	6.4
H5	Leu-Arg	55.4	4.8
**PM7**			
A5	Leu-Val	58.4	7.5

A6	Lys-Ala	55.9	4.9
B1	Lys-Pro	54.8	3.9
B6	Lys-Val	54.4	3.4
B7	Met-Arg	60.1	9.1
B12	Met-His	54.2	3.2
C1	Met-Ile	54.9	3.9
C2	Met-Leu	55.9	4.9
C8	Met-Val	61.3	10.3
D4	Pro-Ala	57.4	6.4
D11	Pro-Pro	54.8	3.9
E1	Ser-Ala	59.5	8.5
E2	Ser-Gly	54.0	3.0
E7	Ser-Pro	60.4	9.4
E8	Ser-Ser	56.4	5.4
E10	Ser-Val	58.4	7.5
E11	Thr-Ala	58.9	7.9
E12	Thr-Arg	54.0	3.0
F5	Thr-Pro	61.3	10.4
**PM8**			
A5	Ala-Ile	54.4	3.5
A7	Ala-Val	62.4	11.5
B6	Ile-Asn	57.9	7.0
B7	Ile-Leu	56.5	5.6
B10	Leu-Pro	55.0	4.1
C3	Met-Thr	56.9	6.0
C11	Pro-Arg	54.2	3.3
D6	Pro-Val	57.0	6.1
D7	Ser-Asn	54.5	3.6
E2	Thr-Ser	55.4	4.5
E6	Val-Ala	64.9	14.0
E7	Val-Gln	55.1	4.2
E10	Val-Met	54.1	3.2
E11	Val-Phe	54.8	3.9
E12	Val-Pro	64.0	13.1
F1	Val-Ser	61.9	11.0

Supp. Table 4) DNA fragments encoding individual SBPs synthesized by Genescript and cloned into expression vectors.

ORF (nucleotides of the gene)[1]	DNA sequence (5'-3')
PA0283 (67 - 999)	GCCACGCAACTGCTGAACGTTTCCTACGACCCGACCCGCGAGCTGTACCAGGCCTACAACGCGGCCTTCATCAAGCACTGGAAGGCCCAGGGCGGCG<br>AGGACCTGACCGTCCAGCAGTCCCACGGCGGCTCCGGCAAGCAGGCCCGCGCGGTGATCGACGGGCTCAAGGCCGACGTGGTGACCCTGGCCCTGGC<br>CGGCGACATCGACGAACTGCACAAGCTCGGCAAGCTGCTGCCGGCCGACTGGCAGGCACGCCTGCCGGACAACAGCACCCCCTACACCTCGACCATC<br>GTGTTCCTGGTGCGCAAGGGCAACCCCAAGGGCATCAAGGACTGGGGCGACCTGACCAAGGAAGGCGTGGAAGTCATCACACCGAACCCGAAGACCT<br>CCGGCGGCGCGCGCTGGAACTTCCTCGCCGCCTGGGCCTGGGCGAAGAAGCAGTACGGCAGCGACGAGAAGGCCAAGGACTACGTGCAGGCGCTGTA<br>CAAGCACGTACCGGTGCTCGACACCGGCGCCCGCGGCTCGACCATCACCTTCGTCAACAACCAGATCGGCGACGTGCTGCTGGCCTGGGAGAACGAG<br>GCGTTCCTGGCGAAGAAGGAACAGGGCGGGGAAAACTTCGAGATCGTCGTACCCTCCATCTCCATCCTCGCCGAACCGCCGGTCGCGGTGGTCGACA<br>AGGTGGTCGAGAAGAAAGGCACCCGCAAGGTCGCCGAGGCCTACCTGCAATACCTGTACAGCGAGGAAGGCCAGCGCATCGCCGCACAGAACTTCTA<br>TCGGCCGCGCAACCAGAAGGTCGCCGCCGAGTTCGCCACGCAGTTCCCGAAACTGAACCTGGTCACCGTCGACTCCGATTTCGGCGGCTGGAAGACG<br>GCCCAGCCGAAGTTCTTCAACGACGGGGGCATCTTCGACCAGATCTACCAGGCGCAATGA
PA0888 (64 - 777)	GCCGACAAGCCGGTGCGCATCGGCATCGAGGCGGCCTACCCGCCGTTCTCGCTGAAGACCCCGGACGGCCAGTTGGCAGGCTTCGACGTGGATATCG<br>GCAATGCCCTCTGCGAAGAGATGAAGGTGCAGTGCAAGTGGGTCGAGCAGGAGTTCGACGGCCTGATCCCGGCACTCAAGGTGCGCAAGATCGATGC<br>GATCCTCTCGTCGATGACCATCACCGACGAGCGCAAGCGTTCGGTCGACTTCACCAACAAGTACTACAACACTCCGGCGCGCTTCGTGATGAAGGAA<br>GGCGCCAGCCTCAACGATCCCAAGGCCGACCTGAAGGGCAAGAAGGCCGGCGTGCTGCGTGGCAGTACCGCCGACCGCTACGCCTCCGCCGAACTGA<br>CCCCGGCCGGCGTCGAAGTGGTGCGCTACAACTCCCAGCAGGAAGCCAACATGGACCTGGTGGCCGGTCGCCTCGACGCGGTGGTCGCCGATTCGGT<br>GAACCTCGAGGACGGCTTCCTCAAGACCGATGCCGGCAAGGGCTACGCCTTCGTCGGTCCGCAACTGACCGATGCCAAGTACTTCGGCGAAGGCGTC<br>GGCATCGCCGTGCGCAAGGGCGATAGCGAGCTGGCCGGCAAGTTCAACGCGGCCATCGACGCGCTGCGCGCCAACGGCAAGTACAAGCAGATCCAGG<br>ACAAGTACTTCAGCTTCGACGTCTACGGTTCGAAC
PA1074 (79 - 1119)	GCCGACACCATCAAGATCGCCCTGGCTGGCCCGGTCACCGGTCCGGTAGCCCAGTACGGCGACATGCAGCGCGCCGGTGCGCTGATGGCAATCGAAC<br>AGATCAACAAGGCAGGCGGCGTGAACGGCGCGCAACTCGAAGGCGTGATCTACGACGACGCCTGCGATCCCAAGCAGGCCGTGGCGGTCGCCAACAA<br>GGTGGTCAACGACGGCGTCAAGTTCGTGGTCGGTCATGTCTGCTCCAGCTCCACCCAACCCGCCACCGACATCTACGAAGACGAAGGCGTGCTGATG<br>ATCACCCCGTCGGCCACCGCCCCGGAAATCACCTCGCGCGGCTACAAGCTGATCTTCCGCACCATCGGCCTGGACAACATGCAGGGCCCGGTGGCCG<br>GCAAGTTCATCGCCGAACGCTACAAGGACAAGACCATCGCGGTACTGCACGACAAGCAGCAGTACGGCGAAGGCATCGCCACCGAGGTGAAGAAGAC<br>CGTGGAAGACGCCGGCATCAAGGTTGCCGTCTTCGAAGGCCTGAACGCCGGCGACAAGGACTTCAACGCGCTGATCAGCAAGCTGAAGAAAGCCGGC<br>GTGCAGTTCGTCTACTTCGGCGGCTACCACCCAGAAATGGGCCTGCTGCTGCGCCAGGCCAAGCAGGCCGGGCTGGACGCGCGCTTCATGGGCCCGG<br>AAGGGGTCGGCAACAGCGAAATCACCGCGATCGCCGGCGACGCTTCGGAAGGCATGCTGGCGACCCTGCCGCGCGCCTTCGAGCAGGATCCGAAGAA<br>CAAGGCCCTGATCGACGCCTTCAAGGCGAAGAACCAGGATCCGAGCGGCATCTTCGTCCTGCCCGCCTACTCCGCGGTCACAGTGATCGCCAAGGGC<br>ATCGAGAAAGCCGGCGAGGCCGATCCGGAGAAGGTCGCCGAGGCCCTGCGCGCCAACACCTTCGAGACTCCCACCGGGAACCTCGGGTTCGACGAGA<br>AGGGCGACCTGAAGAACTTCGACTTCACCGTCTACGAGTGGCACAAGGACGCCACCCGGACCGAGGTCAAG
PA1342 (70 - 906)	GACGAGCTCACCGGCACGCTGAAGAAGATCAAGGAAACCGGCACCATCACCCTCGGTCACCGTGACGCTTCGATTCCCTTCTCCTACCTCGGCACCG<br>AGCCGGGCAAGCCGATCGGCTACTCCCACGACCTGCAACTGAAAGTGGTCGAGGCGGTGAAGAAGGAACTCAACCTCCCCGAGCTGAAGGTCCGCTA<br>TAACCTGGTCACCTCGCAGACCCGCATCCCGCTGGTGCAGAACGGCACCGTGGACATCGAGTGCGGCTCCACCACCAACAACGAAGAGCGCCAGAAG<br>CAGGTCGACTTCTCCGTCGGCATCTTCGAGGTCGGCACCCGCCTGCTGTCGAAGAAGACCGCCAACATCAAGGACTTCGACGACCTCAAGGGCAAGA<br>ACGTGGTGACCACCGCCGGCACCACCTCCGAGCGCCTGCTCAAGGCCATGAACGCGGACAAGAAGATGGGCATGAACATCATCTCCGCCAAGGACCA<br>CGGCGAGTCCTTCATGATGCTCGAATCCGGCCGCGCGGTGGCCTTCATGATGGACGACGCGCTGCTCTACGGCGAAATGGCCAAGGCCAAGAAGCCG<br>GACGACTGGGTCGTCGGCGGCACCCCGCAGTCCTTCGAGATCTACGGCTGCATGGTGCGCAAGGGCGACGCGGCGTTCAAGAAAGTGGTCGACAAGG<br>CCATCACCGATACCTACGCCTCCGGCGAGGTCAACAAGATCTACGACAAGTGGTTCACCCAGCCGATCCCGCCGAAGGGCCTGAACCTCAACTTCCC<br>CATGAGCGAAGAGCTGAAGAAGCTGATCGCCAGCCCGACCGACAAGGCCGCCGAGCAGATG

PA1493 (67 - 999)	GCCCAGCCCCTGCTCAACGTCTCCTACGACGTGATGCGCGACTTCTACAAGGAATACAACCCGGCCTTCCAGAAATACTGGAAAGCGGAGAAAGGCG<br>AGAACATCACCATCCAGATGTCCCATGGCGGTTCCAGCAAGCAGGCGCGCTCGGTGATCGACGGTCTGCCCGCCGACGTCATCACCATGAACCAGGC<br>CACCGACATCGACGCCCTCGCCGACAACGGCGGCCTGGTGCCGAAGGACTGGGCGACCCGCCTGCCGAACAACAGCGCGCCGTTCACCTCGGCCACC<br>GTGTTCATCGTTCGCAAGGGCAACCCCAAGGCCCTGAAGGACTGGCCGGACCTGCTCAAGGACGGCGTCCAGGTAGTGGTGCCGAACCCCAAGACCT<br>CCGGCAACGGCCGCTATACCTATCTCTCCGCCTGGGGCTACGTGCTGAAGAACGGCGGTGACGAGAACAAGGCCAAGGAATTCGTCGGCAAGCTGTT<br>CAAGCAGGTACCGGTGCTCGACACCGGCGGCCGCGCCGCCACCACTACCTTCATGCAGAACCAGATCGGCGACGTACTGGTGACCTTCGAGAACGAA<br>GCCGAGATGATCGCCCGCGAATTCGGCCGAGGCGGCTTCGAGGTGGTCTACCCGAGCGTGTCCGCCGAGGCCGAGCCCCCGTGGCGGTGGTCGACA<br>AGGTGGTCGAGAAGAAAGGCAGCCGCGCCCAGGCCGAGGCCTACCTGAAGTACCTGTGGTCGGACGAGGGCCAGACCATCGCCGCCAACAACTACCT<br>GCGCCCGCGCAACCCGGAGATCCTCGCCAAGTTCGCCGACCGCTTCCCGAAAGTCGACTTCTTCTCGGTGGAGAAGACCTTCGGCGACTGGCGCAGC<br>GTGCAGAAGACCCACTTCATCGACGGCGGCGTATTCGACCAGATCTACAGCCCCAACTGA
PA1863 (70 - 756)	GACGAGGTGCAGGTCGCCGTCGCGGCCAACTTCACCGCGCCGATCCAGGCCATCGCCAAGGAATTCGAGAAAGACACCGGGCACAGGCTGGTCGCCG<br>CCTACGGCGCCACCGGCCAGTTCTATACGCAGATCAAGAACGGCGCGCCGTTCCAGGTGTTCCTCTCCGCCGACGACAGCACTCCGGCGAAACTGGA<br>GCAGGAAGGCGAGGTCGTGCCCGGCTCGCGCTTCACCTATGCCATCGGCACCCTGGCACTCTGGTCGCCCAAGGCCGGCTATGTCGACGCCGAGGGC<br>GAGGTGCTGAAGAGCGGCAGCTTCAGGCACCTGTCCATCGCCAACCCGAAGACCGCGCCCTACGGCCTCGCCGCCACCCAGGCGATGGACAAGCTCG<br>GCCTCGCCGCCACGCTCGGGCCGAAGCTGGTGGAAGGCCAGAACATCAGCCAGGCCTACCAGTTCGTTTCCAGCGGCAACGCCGAACTGGGCTTCGT<br>CGCTCTGTCGCAGATCTACAAGGATGGTAAAGTCGCAACCGGCTCGGCCTGGATCGTTCCCACCGAGCTGCACGACCCGATCCGCCAGGACGCGGTC<br>ATCCTCAACAAGGGCAAGGACAACGCCGCCGCCAAGGCCCTGGTCGACTACCTGAAGGGCGCCAAGGCCGCCGCGCTGATCAAGTCCTACGGCTACG<br>AACTCTAA
PA1946 (88 - 960)	GAAACTCCCGAGAAGCCCAGGATCGCGCTGGTGATGAAGTCGCTGGCGAACGAGTTCTTCCTGACCATGGAGGACGGCGCCAAGGCCTACCAGAAGG<br>AGCATGCCGACCGGTTCGAACTGGTTTCCAACGGCATCAAGGACGAGACCGATACCTCCAGCCAGATCCGCATCGTCGAGCAGATGATCGTTTCCGG<br>GGTCGACGCACTGGTGATCGCGCCGGCCGATTCCAAGGCGCTGGTGCCGGTGGTGAAGAAAGCGCTGGACGCCGGCATCGTGGTGGTCAACATCGAC<br>AACCGCTTCGACCCGCAGGTGCTGCAGGCGAAGAAGATCGGCGTGCCCTTCGTCGGGCCCGACAACCGCAAGGGTGCGCGGCTGGTCGGCGAGTACC<br>TGGCGAAGCGACTGAAGGTCGGCGACGAGGTCGGCATCATCGAAGGGGTCTCCACGACCACCAATGCCCAGCAGCGCACCGCCGGTTTCAAGGATGC<br>GATGGACGCGGCGGGGATGAAGATCGTCTCCTTGCAGTCGGGCAACTGGGAAATCGAGAAGGGCAACGCGGTGGCCTCGGCGATGCTCAACGAGCAT<br>CCTGACCTCAAGGCGCTGCTCGCCGGCAACGACAGCATGGCCCTCGGCGCGGTATCGGCGGTGCGCGCGGCGGGCCGCGCCGGGCAGGTGAAGGTGG<br>TCGGCTACGACAATATCCAGGCGATCAAGCCGATGCTCAAGGATGGCCGGGTGCTGGCCACTGCCGACCAGTTCGCCGCGAAGCAGGCGGTGTTCGG<br>TATCCAGACCGCGCTCAAGCTGCTCGCCGGGCAAACCCCGGAACATGAGAAGGACGGCGTGGTCGAGACGCCTGTCGAGTTGGTGACCGCGCCCTGA
PA2204 (74 - 807)	GACCGCCTGGAGGACATCCGCAAGGCCGGCGTATTGCGCGTCGCGTCCTTCGACAGCAACCCGCCGTTCGGCTTCGTCGACGCCAAGAGCAAGCAGA<br>TCGAAGGCCTCGACGTCGACTATGCCAAGGCCCTGGCCGACAAGCTGGGCGTCCGGCTGCAAGTGCTGCCGACCAACCCGGCCAACCGCATCCCGCT<br>GCTGACGGCGAACAAGGTCGACCTGGTCCTGGCCAACTTCACCATCACCCCGGAGCGTGCGCAGCAGGTGGACTTCAGCATCCCCTACTTCTCCTCC<br>GGCCAGCAGTTCATCGTCAAGAAAGGCACCCTGACCTCGCCGGAAGTCCTCAACAAGTGGCGGGTCGGCGTGGACAAGGGCACGGTCAACGAAGGCG<br>TGCTGCGCGAGAAGTTCCCCGGCGCCAAGGTCATCGCCTACGACGACACGCCCTTCGCTTTCACCGCCCTGCGCAACGGCCAGGTCCAGGCCATCAC<br>CCAGGACGGTCCGAAGCTGATCGGCCTGCTGGCCAACGTGCCCGACCGCGACAAGTACGAAGTGCCGCCCTTCACCATTTCCAACGACCTGATCGGC<br>GTCGGTATTCCCAAGGGCGAGAAGGCCCTGACCGAGTTCGTCGACAAGAGCCTGCGCGAGCTGGAGCAGGACGGCCAGGCGCAGAAGATCTACGACA<br>CCTGGTTCGGTCCGCAGACCAAGACCCCGCTGGCGCGCCTGTACAAGATCGGCGACAAGAGCTGA
PA2338 (67 - 1308)	GCCGAGACCCTGACCATCGCCACGGTCAACAACAACGACATGATCCGCATGCAACGCCTGTCCAAGGTGTTCGAGGAAAGCCACCCGGACATCGCGC<br>TGAAATGGGTGGTGCTCGAAGAGAACGTCCTGCGCCAGCGCCTGACCACCGACATCGCCACCCAGGGCGGACAGTTCGACCTGCTCACCATCGGCAT<br>GTACGAAGCCGCGCTTTGGGGCGCCAAGGGCTGGCTGGAGCCGATGAGCGGGCTGCCCGCCGACTACGCCCTGGACGACCTGTTGCCCTCGGTGCGC<br>GACGGTCTCTCCGTCAAGGGCACGCTGTACGCCCTGCCGTTCTACGCCGAAGCCTCGATCACCTACTACCGCAAGGACCTGTTCCAGCAGGCCGGGC<br>TGCGGATGCCCGAGCAACCGACCTGGACCCAGCTCGGCGAGTTCGCCGCCAGGCTCAACCGCCCGGACCAGGGCCAGTACGGTATCTGCCTGCGCGG<br>CAAGGCCGGCTGGGGCGAGAACATGGCGTTGATCGGCACCCTGGCCAACGCCTTCGGCGCACGCTGGTTCGACGAGCGATGGCAGCCCGAATTCAGC<br>GGCGGCGAATGGAAGAAGGCTCTCGACTTCTATGTCTCCACCCTCAAGCGGTACGGCCCGCCCGGCGCCTCCAGCAACGGATTCAACGAGAACCTGG<br>CGCTGTTCAACAGCGGCAAGTGCGCGATCTGGGTGGACGCCAGCGTCGCCGGTTCGTTCGTCACCGACAAGAGCCAGAGCAAGGTCGCCGACGCCAC<br>CGGCTTCGCCTTCGCCCCCGGGAAGTCACCGACAAGGGCGCCTCCTGGCTGTATTCCTGGGCCCTGGCGATTCCCGCCAGCTCGCGCGCCAAGGAC<br>GCGGCGAAGGCCTTCGCCACCTGGGCCACCTCGCAGGCGTACGGCAAGCTGGTGGCGGACAGGGAAGGCGTAGCCAACGTGCCGCCCGGCACCCGCG

	CCTCGACCTACAGCGAGGCCTACCTGGCCGCCGCGCCGTTCGCGCGGGTCACCCTGGAATCGCTGAAGCGCGTCGATCCCAACCATCCGACGCTGAA<br>GCCGGTGCCCTATGTCGGCATCCAGTTGGTCACCATCCCCGAGTTCCAGGCCATCGGCACCCAGGTCGGCAAGCTGTTCTCCGCCGCCCTCACCGGG<br>CAGATGAGCAGCGACCAGGCGCTCGCCGCCGCGCAGCAGAGCACCGCCCGCGAGATGAAGCGCGCCGGCTATCCGAAG
PA2592<br>(58 - 1101)	GCGGACAGTGCATGGGCTGCGCAAACCAGCGTCCATCTTTACAACTGGTATGACTTCATCGCCCCGGAAACGCCCAAGGCTTTCCAGAAGGAAACCG<br>GCACCCGTGTCGTCCTCGACACCTTCGACAGCGCCGAGACCGCGCAGGGCAAGCTGATGGTCGGCCGCTCCGGCTACGACGTGGTGGTGATCACCTC<br>CAACATCCTGCCCGGGCTGATCAAGGCGGGCGTCCTCCAGGAACTCGACCGCGACCGGCTCCCCCACTGGAAGAACCTCGACGCGGACATCCTCGGG<br>AAGCTTCAGGCCAACGATCCCGGCAATCGCTATGCCGTACCTTATCTCTGGGGAACCACCGGGATCGCCTACGATGTGGACAAGGTCCGCAAGCTGC<br>TCGGCCCCGACGCGCCGGTCGACTCCTGGGACCTGGTCTTCAAGGAGGAGAACATCTCCCGCCTCAGCCAGTGCGGCGTGGCCACGCTGGACTCCTC<br>CACCGAGCTGGTGTCCATCGCCCTCAACTACCTGGGCCTGCCGCACAACAGCCAGAATCCCGAGGACTACCAGAAAGCCCAGGAACTGTTGCTGAAG<br>GTTCGCCCCTACATTCGCTATTTCGACTCCTCCAGAGTCGACACCGATCTCTCCAACGGCAACGTCTGCGTGGTGGTCGGCTGGCAGGGCACGGCCT<br>ACATGGCCCAGGTCAACAACGAACAGGCCGGGAACGGTCGCCATATCGCCTACAGCATTCCCCGGGAAGGCTCGCTGGTCTGGGCCGAGAACATGGT<br>GCTGCTCAAGGATGCACCGCATCCGCAGCAGGGTTATGCGCTGATCGACTACCTGCTGCGTCCGGAGGTCATCGCCAGGACCTCCAACTACGTGGGC<br>TATCCGAATGGCAACCAGGCGGCGCTGCCGCTGGTAGAGCGGAAACTGCGGGAAAACCCGGCGGTTTACCTGAGCAAGGAAACCATGGCGACCCTCT<br>TCCCGCTGGAAACCCTGCCACTGAAGGTCGAGAGAATCCGTACCCGGGTCTGGAGCCGGGTCAAGACCGGGAGC
PA3610<br>(61 - 1062)	GCCGAGAAGCTGTATCTGTTCAACTGGAACGACTACATCGCCGAGGACACGCTGAAACGCTTCGAGCAGCAATGTGGCTGCGAGCTGGTGCAGGAGT<br>TCTATTCCGGCACCGAGGAAATGATGGCCAAGCTGGCCGCCGGGGCCAGCGGCTACGACGTGATCATCCCGACCCAGAACGCGGTCGAGGCGCTGAT<br>CCGCAAGGGCGACCTGCTGGAGCTGGACAAGAGTCGCCTGGCGAACCTGAGCAACGAGGCCGCGGGCTACCTCGACAAGGATTTCGACAAGGGCAAC<br>CGCTATTCCCTGCCCTACGCCTTCACCACCACCCTGGTCGGCTACAACAAGACCGAGCTGGACAAGCTGGGCATCGACCCCGCCGACTGGTCGGTGA<br>TCTTCGACCCGGCGGTCCTGGAGAAGATCAAGGGCAGGGTCACGGTGATGGACGACCCGCAGGAACTGTTCGGCGCCGCCCTGAAGTACCTCGGTCA<br>CTCCGCCAACGACACCGACCCGCAGCACTGGAAGGAAGCCCAGGCGCTGATCCTCGCGGCCAAGCCGTACTGGGCCGCGTTCAACTCGTCGAGCTAT<br>ATCAAGGAGCTGACCCTGGGCAACATCTGGGTCGCCCATGGCTACTCCAGCGACATGTACCAGGCCAGGGCCGACGCGGAGGCTGCCGGCCGCGCCT<br>TCAAGGTCGACTTCGCCCTGCCCCGCCAAGGTGCGGTGCTGGCCATCGACAACATGGTGATCCACAAGGGCTCGAAGAACCCTGACCTGGCCTACCG<br>CTTCATCGACTTCATGCTCGACGGTCGGAACGCTTCCGAGCTGACCAACCAGATCGGTACCGGCACGCCCAACGCCGCCGCCCTGCCCTTCATCAAG<br>CCGGAAATCAAGACGCTCGCCGCCCTGTTCCCGGACGCCACCACCCAGGCCAGGCTGGAGCCGCTGAAGGACCTGAACTCGCGCCAGCGACGCGCCC<br>TGAACAAGCTCTGGACAGAGATCAAGCTGCGC
PA3889<br>(70 - 936)	GAAACCCTGCGCATCGGCGGCAAGACCTTCACCGAGCAGCGCATCCTCACCGCCATCACCGCGCAGTTCCTGCAGAAGCGCGGCTACGACGTGACGG<br>TCACCACCGGCCTGGGCAGCACCCTGGCCCGCGCCGCCCAGGAAAGCGGCCAACTGGATATCGTCTGGGAGTACACCGGCTCGTCGCTGATCGTCTA<br>CAACCACATCGACGAGAAGCTCGACGCCGCGGCTTCCTACCGCCGGGTCAAGCAACTCGACGAAGCCCAGGGCCTGGTCTGGCTGAAGCCGACCCGC<br>TTCAACAACACCTATGCCCTGGCCATGCCCGAGGAACAGGCCGAGCACCTGGGCATCCAGAGCGTCAGCGATCTCGCGCGGGTGCTCGCCGAACAGC<br>AGGAGGCCGAGCCCGGGAGCACCCACCTGTTCGCCATGGACCCGGAGTTCGCCGGCCGCCCCGACGGCCTCGGCCCCATGAGCGAGCTGTACGGCCT<br>GCACTTCACCCGCAACGACATCCGCCAGATGGACGCCGGGCTGGTCTACACCGCGCTGAAGAACCGCCAGGTGTTCCTCGGCCTCGTCTACACCACC<br>GACGGCCGCCTGAAGGATTTCAAGCTGCGCGTGCTGAAGGACGACAAGCAGTACTTCCCCTTCTACAACGCCGCCCCGGTGGTGCGTAAGGAGGTCA<br>TGCAGCGCCATCCGGAGTTCGCCACGCTGTTCGATCCGATCATCGAGCGGCTCGACGACGCCACCATGCAGGCGCTCAACGCGCGGGTCGATATCGA<br>GCAGCAGACGCCACAGAAGGTCGCCGCCGACTTCCTCCGCGAACACCACCTGCTCGACGACGGCCAGGCCGGCCAGGGAGGTAGCCAGTAA
PA4497<br>(73 - 1596)	CAGCCGAAGACCCTGGCGGTGTGCACCGAAGCGGCGCCGGAAGGGTTCGATCCGGCCCGCTATACCTCCGGCTACACCTTCGACGCCTCAGCCCATC<br>CGCTGTACAACGCGCTGGCCGCGTTCGCTCCGGGCAGCGCCACGGTGATCCCGGCCCTGGCGGAAAGCTGGGACGTCTCCGCTGACGGCCTCGTCTA<br>CACCTTCCGCCTGCGCCAAGGCGTGAAATTCCACAGCACCGACTACTTCAAGCCAACCCGCGAATTCGACGCCGACGACGTGCTGTTCAGCTTCCAG<br>CGCATGCTCGATCCGCAGCACCCCGCCCACGACCTTTCGCCCAGCGGCTACCCCTACGCCGACGCCATGCAACTGCGCGACATCATCGAGCGCATCG<br>AGAAGATCGACGAACATCAGGTGCGCTTCGTCCTCAAGCACCCGGAGGCGCCGTTCCTTGCCGACCTGGCCATGCCGTTCGGCTCGATCCTCTCCGC<br>CGAATATGCCGGCCAGTTGATCGCCAGGGGCAAGGGCGACGAACTCAACAGCAAGCCGATCGGCACCGGCCCGTTCGTCTTCACCCGCTACCGCAAG<br>GATGCCCAGGTGCGCTACGCAGCCAACCCCGACTACTGGAAGGGCAAGCCGGCCATCGACCACCTGGTGCTGGCGATCACCCTCGACCCCAACGTGC<br>GCGTGCAGCGCCTGCGCCGCAACGAATGCCAGATCGCCCTGACGCCCAAGCCCGAAGACGTCGCCGCGTTACGCCAGGACCCGCAACTGACGGTGCT<br>GGAAGAAGCGGCGATGATCACCTCACACGCCGCGATCAACACCCGGCACGAACCGTTCGACGACCCGCGCGTGCGCCGGGCGATCGCCATGGGCTTC<br>AACAAGTCGTCCTACCTGAAGATCGTCTTCGGCGACCAGGCCCGCCCGGCCATCGGCCCCTACCCGCCGATGCTGCTCGGCTACGACGACAGCATCC<br>GCGACTGGCCCTACGACCCCGAACGGGCCAAGGCCCTGCTGAAGGAAGCCGGCGTCACCCCGGACACACCCCTGAACCTCTACATCAGCACCGGCAG

	CGGCCCCGGCGGCAACCCGGCGCGAGTGGCGCAACTGATCCAGTCCGACCTGGCCGCCATCGGCATCCGGGTGAACATCCGCCAGTTCGAGTGGGGC<br>GAGATGGTCAAGCGCACCAAGGCCGGCGAACACGACATGATGCTCTACAGCTGGATCGGCGATAACGGCGACCCGGACAACTTCCTCACCCACAACC<br>TCGGCTGCGCCTCGGTGGAGTCCGGGGAAAATCGCGCGCGCTGGTGCGACAAGGGCTTCGACGAAGCCATCCGCAAGGCGCGCATGAGCAACGACGA<br>GAGCCAACGGGTAGCGCTGTACAAGGAAGCGCAGCGGATCTTCCACGAGCAGATGCCCTGGTTGCCGCTGGCGCATCCTTTGATGTTCGACGCGCAA<br>CGCAAGAACGTCAGCGGCTACCGCATGAGCCCGATGTCGGCGCGGGACTTTTCGCGGGTGAAGCTGGAC
PA4500<br>(73 - 1599)	GCCAGCAACCTGGTGTTCTGCTCGGAGGGCAGCCCGGCCGGCTTCGATCCCGCCCAGTACACCACCGGCACCGACTACGACGCGACCTCGGTCACCC<br>TGTTCAACCGCCTCGTCCAGTTCGAGCGCGGCGGCACCCGGGCGATCCCGGCCCTGGCGGAAAGCTGGGACATCGGCGACGACGGCAAGACCTACAC<br>CTTCCACCTGCGCAAGGGTGTGAAGTTCCACAGCACCGACTACTTCAAGCCGACCCGCGAATTCAACGCCGACGATGTGCTGTTCACCTTCCAGCGC<br>ATGCTCGACAAGAATCATCCGTTCCGCAAGGCCTACCCCACCGAGTTTCCCTACTTCACCGACATGGGCCTGGACAAGAACATCGCCAGGGTCGAGA<br>AGCTCGACGAGCACAGGGTGAAGTTCACCCTCAACGAGGTCGACGCCGCCTTCATCCAGAACCTGGCGATGGACGTCGCCTCGATCCAGTCCGCCGA<br>GTACGCCGGGCAGTTGCTCGAGGCCGGCAAGCCACAGCAGATCAACCAGAAGCCGATCGGCACCGGGCCTTTCATCCTGAGCCGCTACCAGAAGGAT<br>GCGCAGATCCGCTTCAAGGGCAACAAGGACTACTGGAAACCCGAAGACGTGAAGATCGACAACCTGATCTTCTCGATCAACACCGACGCCGCGGTGC<br>GCGCGCAGAAGCTCAAGGCCGGCGAGTGCCAGATCACCCTCAACCCACGCCCCGCCGATCTCAAGGCCCTGCAGGAAGCGGCGAACCTGAAGGTGCC<br>CTCGCAGCCAGGCTTCAACCTCGGCTACATCGCCTACAACGTCACCCACAAGCCGTTCGACCAGCTCGAAGTACGCCAGGCGCTAGACATGGCGGTG<br>AACAAGCAGGCCATCATCGACGCCGTCTACCAGGGTGCCGGGCAACTGGCGGTGAACGGCATGCCGCCGACCCAGTGGTCCTATGATGAAACCATCA<br>AGGACGCCCCGTTCGATCCGGCCAAGGCCCGGGAACTGCTGAAGAAGGCCGGCGTCGCCGAGGGCACCGAGATCACCCTGTGGGCGATGCCGGTGCA<br>GCGTCCCTACAACCCCAACGCCAAGCTGATGGCCGAGATGATCCAGGCCGACTGGGCGAAGATCGGCATCAAGGCCAGGATCGTCAGCTACGAATGG<br>GGCGAGTACATCAAGCGCGCCCACGCCGGCGAGCACGACGCCATGCTGTTCGGCTGGACCGGCGACAACGGCGATCCGGACAACTGGCTGGCGACCC<br>TCTACGGCTGCGACTCGATCAACGGCAACAACGTATCGAAATGGTGCGACGCGGCCTACGACAAGCTGGTCAAGGCTGCCAAGCGGGTCTCCGACCA<br>GGACAAGCGCAGCGAGCTGTACAAGCAGGCCCAGCACATCCTCAAGGAGCAGGTGCCGATCACCCCGATCGCCCACTCCACCGTCTACCAGCCGATG<br>AGCAAGTCGGTCCACGGCTTCAAGATCAGTCCCTTCTCGCGGAACGCCTTCTATGGCGTAGCCAACCAGCCC
PA4687<br>(82 - 1008)	GATCCCGTCACCCTTACCCTCTACAATGGCCAGCACGCCGCCACCGGTATCGCCATCGCCAAGGCCTTCCAGGACAAGACCGGCATCCAGGTGAAGA<br>TCCGCAAGGGCGGCGATGGCCAGCTCGCCAGCCAGATCACCGAGGAAGGCGCACGCTCGCCGGCCGACGTGCTCTACACCGAGGAATCGCCACCGTT<br>GATCCGCCTGGCCAGCGCCGGCCTGCTGGCCAAGCTGGAGCCAGAGACCCTGGCCCTGGTCGAGCCGGAGCACGCCGGCGGCAACGGCGACTGGATC<br>GGCATCACCGCCCGCACCCGGGTACTGGCCTACAACCCGAAGAAGATCGACGAGAAGGATCTGCCGAAGAGCCTGATGGATCTTTCCGATCCGTCCT<br>GGTCCGGCCGTTTCGGCTTCGTGCCCACCAGTGGCGCCTTCCTCGAACAGGTCGCCGCCGTGATCAAGCTCAAGGGCCAGGAGGAAGCCGAGGATTG<br>GCTGACCGGCCTGAAGGCCTTCGGCTCGATCTACACCAACAACGTCACCGCCATGAAAGCCGTGGAGAACGGCGAGGTCGACATGGCGCTGATCAAC<br>AACTACTACTGGTACACCCTGAAGAAGGAAAAGGGCGAGTTGAACTCGCGCCTGCACTACTTCGGCAACCAGGACCCGGGCGCGCTGGTCACCGTCT<br>CCGGCGCCGCGGTGCTCAAGTCCAGCAAGCACCCCAGGGAAGCCCAGCAGTTCGTCGCCTTCATGCTCAGCGAGGAAGGCCAGAAGGCGATCCTCAG<br>CCAGTCCGCCGAGTACCCGATGCGCAAGGGGATGCAGGCCGATCCGGCGCTGAAGCCGTTCGCCGAACTGGACCCACCGAAACTGACCCCGGCCGAT<br>CTGGGTGAGGCCAGCGAGGCCCTCAGCCTCGAACGCGACGTTGGCCTGAATTGA
PA4913<br>(82 - 1122)	GACGTCGTGATCGGCGTGGCCGGACCGCATACCGGCGCCAACGCCTCCTTCGGCGAGCAATACTGGCGCGGCGCGTCCCAGGCGGCGGAAGACATCA<br>ACGCGGCGGGTGGGATCAACGGCGAGAAGATCAAGCTGGTCAAGGCCGACGACGCCTGCGAACCGAAGCAGGCCGTGGCCGTGGCCAACCGCCTGGT<br>GGACCAGGACAAGGCCATCGCCGTGGTCGGCCATTTCTGCTCTTCTTCGACCATCCCCGCATCCGAGGTCTACGACGAGGCCGGGATCATCGCCATC<br>ACCCCCGGTTCCACCAACCCGCAGGTCACCGAGCGCGGACTCTCCGGGATGTTCCGCATGTGCGGCCGCGACGACCAGCAGGGCGTGGTCGCCGGCG<br>ACTATATCGTCAACGTGCTCAAGGCCAAGAAGGTCGCGGTGATCCATGACAAGGACACCTACGGCCAGGGCCTGGCGGACGCCACCAGGGCGCAACT<br>GAACAAGCTCGGCGTGAAAGAGGTTCTCTACGAGGGCCTGACTCGCGGCGAGAAGGACTTCAACGCGCTGGTCACCAAGATCCGCGCCTCCGGTGCC<br>GAGGTCGTCTACTTCGGCGGCCTGCATCCGGAAGCCGGTCCGCTGGTACGGCAGATGCGTGAACAGGGACTAACCGCCAGGTTCATGTCCGACGACG<br>GCGTGGTCACCGACGAACTGGCGACCACCGCCGGCGGTCCGCAGTACGTCAAGGGCGTGCTGATGACCTTCGGCGCCGACCCGCGGCTGATCCCCGA<br>CGGCAAGGCGGTGGTGGAGAAGTTCCGTGCCGGCGGTTTCGAGCCGGAAGGCTACACCCTCTACTCCTACGCCTCGATCCAGTCCCTGGCGGCGGCC<br>TTCAACGGCGCCGGCGCCAACGATCCGGCCAAGGCCGCCGAGTGGCTGAAGTCGCATCCGGTGCAGACCGTGATGGGCAAGAAGGAATGGGACAAGA<br>AGGGCGACCTGAAAGTCTCCGACTACGTGGTCTACGAGTGGGACGACAAGGGCAAGTACCACCAGTTGCCC

[1] The initial nucleotides of the gene were not included into the proteins since they encode signal peptides. In all cases the last nucleotide is that at the end of the gene.

Supp. Table 5) Experimental conditions for protein overexpression, purification and analysis.

ORF	Restriction sites for cloning	*E. coli* culture conditions		Protein purification		Analysis buffer
		Temperature before/after induction (ºC)	IPTG conc. (mM)	Buffer A	Buffer B	
PA0222[1,3]	NdeI, EcoRI	37/18	0.1	50 mM NaH_2PO_4, 150 mM NaCl, 20 mM imidazole, pH 7.5	50 mM NaH_2PO_4, 150 mM NaCl, 500 mM imidazole, pH 7.5	50 mM HEPES, 150 mM NaCl, pH 7.5
PA0283[1,3]	NdeI, BamHI	37/18	0.1	50 mM NaH_2PO_4, 150 mM NaCl, 20 mM imidazole, pH 7.5	50 mM NaH_2PO_4, 150 mM NaCl, 500 mM imidazole, pH 7.5	50 mM HEPES, 150 mM NaCl, 10 % (v/v) glycerol, pH 7.5
PA0888[2,3]	NcoI, NotI	30/18	0.1	20 mM Tris/HCl, 0,1 mM EDTA, 200 mM NaCl, 10 mM imidazole, 5 % (v/v) glycerol, pH 8.0	20 mM Tris/HCl, 0,1 mM EDTA, 200 mM NaCl, 500 mM imidazole, 5 % (v/v) glycerol, pH 8.0	5 mM Tris/HCl, 5 mM PIPES, 5 mM MES, 150 mM NaCl, 10 % (v/v) glycerol, pH 7.0 (4 dialyses)
PA1074[2,3]	EcoRI, XhoI	30/18	0.1	50 mM Tris/HCl, 500 mM NaCl, 1 mM DTT[4], 10 mM imidazole, 10 % (v/v) glycerol pH7.5	50 mM Tris/HCl, 500 mM NaCl, 1 mM DTT, 500 mM, imidazole, 10 % (v/v) glycerol pH 7.5	50 mM HEPES, 100 mM NaCl, 1 mM DTT, 10 % (v/v) glycerol, pH 7.5
PA1342[2,3]	NcoI, NotI	30/18	0.1	20 mM Tris/HCl, 200 mM NaCl, 1 mM EDTA, 10 mM imidazole, 5 % (v/v) glycerol, pH 8.0	20 mM Tris/HCl, 200 mM NaCl, 1 mM EDTA, 1 M imidazole, 5 % (v/v) glycerol, pH 8.0	Dialysed into 50 mM HEPES, 150 mM NaCl, 10 % (v/v) glycerol, 6 M GdnHCl[5], pH 6.5, then refolded by 2 dialysis steps into 10 mM Tris/HCl, pH 6.5
PA1493[1,3]	NdeI, BamHI	37/18	0.1	50 mM NaH_2PO_4, 150 mM NaCl, 20 mM imidazole, pH 7.5	50 mM NaH_2PO_4, 150 mM NaCl, 500 mM imidazole, pH 7.5	50 mM HEPES, 150 mM NaCl, 10 % (v/v) glycerol, pH 7.5
PA1863[1,6]	NdeI, BamHI	30/18	0.5	20 mM Tris/HCl, 0,1 mM EDTA, 200 mM NaCl, 10 mM imidazole, 5 % (v/v)	20 mM Tris/HCl, 0,1 mM EDTA, 200 mM NaCl, 500 mM imidazole, 5 % (v/v)	5 mM Tris/HCl, 5 mM PIPES, 5 mM MES, 150 mM NaCl, 10 %

				glycerol, pH 8.0	glycerol, pH 8.0	(v/v) glycerol, pH 7.4
PA1946[1,3]	NdeI, BamHI	30/18	0.5	20 mM Tris/HCl, 0,1 mM EDTA, 200 mM NaCl, 10 mM imidazole, 5 % (v/v) glycerol, pH 7.0	20 mM Tris/HCl, 0,1 mM EDTA, 200 mM NaCl, 500 mM imidazole, 5 % (v/v) glycerol, pH 7.0	5 mM Tris/HCl, 5 mM PIPES, 5 mM MES, 150 mM NaCl, 10 % (v/v) glycerol, pH 7.0
PA2204[1,3]	NdeI, BamHI	30/18	0.1	20 mM Tris/HCl, 200 mM NaCl, 1 mM EDTA, 10 mM imidazole, and 5 % (v/v) glycerol, pH 8.0	20 mM Tris/HCl, 200 mM NaCl, 1 mM EDTA, 1 M imidazole, 5 % (v/v) glycerol, pH 8.0	Dialysed into 50 mM HEPES, 150 mM NaCl, 10 % (v/v) glycerol, 6 M GdnHCl, pH 6.5, then refolded by two dialysis steps into 10 mM Tris/HCl, pH 6.5
PA2338[2,6]	NcoI, XhoI	30/18	0.5 mM	20 mM Tris/HCl, 200 mM NaCl, 1 mM EDTA, 10 mM imidazole, 6 M GdnHCl, 5 % (v/v) glycerol, pH 8.0	20 mM Tris/HCl, 200 mM NaCl, 1 mM EDTA, 500 mM imidazole, 6 M GdnHCl, 5 % (v/v) glycerol, pH 8.0	5 mM Tris/HCl, 5 mM PIPES, 5 mM MES, 150 mM NaCl, 10 % (v/v) glycerol, pH 6.4
PA2592[2,3]	BamHI, NotI	30/18	0.5mM	20 mM Tris/HCl, 200 mM NaCl, 1 mM EDTA, 10 mM imidazole, 5 % (v/v) glycerol, 6 M GdnHCl, pH 8.0	20 mM Tris/HCl, 200 mM NaCl, 1 mM EDTA, 500 mM imidazole, 5 % (v/v) glycerol, 6 M GdnHCl, pH 8.0	5 mM Tris/HCl, 5 mM PIPES, 5 mM MES, 150 mM NaCl, 10 % (v/v) glycerol, pH 7.4
PA3610[2,3]	BamHI, NotI	30/18	0.1 mM	20 mM Tris/HCl, 200 mM NaCl, 1 mM EDTA, 10 mM imidazole, 5 % (v/v) glycerol, pH 8.0	20 mM Tris/HCl, 200 mM NaCl, 1 mM EDTA, 1 M imidazole, 5 % (v/v) glycerol, pH 8.0	Dialysed into 50 mM HEPES, 150 mM NaCl, 10 % (v/v) glycerol, 6 M GdnHCl, pH 6.5, then refolded by two dialyses into 10 mM Tris/HCl, pH 6.5
PA3889[1,3]	NdeI, BamHI	30/18	0.1 mM	20 mM Tris/HCl, 0,1 mM EDTA, 200 mM NaCl, 10 mM imidazole, 5 % (v/v) glycerol, pH 7.0	20 mM Tris/HCl, 0,1 mM EDTA, 200 mM NaCl, 500 mM imidazole, 5 % (v/v) glycerol, pH 7.0	5 mM Tris/HCl, 5 mM PIPES, 5 mM MES, 150 mM NaCl, 10 % (v/v) glycerol, pH 7.0
PA4497[2,3]	BamHI, NotI	30/30	No IPTG	20 mM Tris/HCl, 0,1 mM EDTA,200 mM NaCl, 10 mM imidazole, 5 % (v/v) glycerol, pH 8.0	20 mM Tris/HCl, 0,1 mM EDTA, 200 mM NaCl, 500 mM imidazole, 5 % (v/v) glycerol, pH 8.0	5 mM Tris/HCl, 5 mM PIPES, 5 mM MES, 150 mM NaCl, 10 % (v/v) glycerol, pH7.4
PA4500[2,3]	NcoI, NotI	30/30	No IPTG	20 mM Tris/HCl, 0,1 mM	20 mM Tris/HCl, 0,1 mM	5 mM Tris/HCl, 5 mM

				EDTA, 200 mM NaCl, 10 mM imidazole, 5 % (v/v) glycerol, pH 8.0	EDTA, 200 mM NaCl, 500 mM imidazole, 5 % (v/v) glycerol, pH 8.0	PIPES, 5 mM MES, 150 mM NaCl, 10 % (v/v) glycerol, pH7.4
PA4687[1,3]	NdeI, BamHI	30/18	0.1 mM	20 mM Tris/HCl, 200 mM NaCl, 1 mM EDTA, 10 mM imidazole, 5 % (v/v) glycerol, pH 8.0	20 mM Tris/HCl, 200 mM NaCl, 1 mM EDTA, 1 M imidazole, 5 % (v/v) glycerol, pH 8.0	Dialysed into 80 mM KCl, 30 mM Na/citrate, 20 mM M, 350 µM K_3PO_4, 6 M GdnHCl, pH 6.5 then refolded by two dialyses into 20 mM MES, 350 µM K_3PO_4, 80 mM KCl, 30 mM Na/citrate, pH 6.5
PA4913[2,3]	NcoI, XhoI	30/18	0.1 mM	50 mM NaH_2PO_4, 150 mM NaCl, 10 mM imidazole, 5 % (v/v) glycerol, 1 mM DTT, pH 8.5	50 mM NaH_2PO_4, 150 mM NaCl 500 mM imidazole, 5 % (v/v) glycerol, 1 mM DTT pH 8.5	50 mM HEPES, 150 mM NaCl, 10 % (v/v) glycerol (pH 7.5)

[1] pET-28b(+) expression plasmid (Novagen)
[2] pET-22b(+) expression plasmid (Novagen)
[3] Expressed in *E. coli* BL21 (DE3) [4]
[4] DTT: dithiothreitol
[5] GdnHCl: Guanidine hydrochloride
[6] Expressed in *E. coli* BL21-AI (Invitrogen), protein induction was performed with 0.2 % (w/v) L-arabinose in addition to the IPTG concentration indicated

References

1. Combet, C.; Blanchet, C.; Geourjon, C.; Deleage, G., NPS@: network protein sequence analysis. *Trends Biochem Sci* **2000,** 25, 147-50.
2. Elbourne, L.D.; Tetu, S.G.; Hassan, K.A.; Paulsen, I.T., TransportDB 2.0: a database for exploring membrane transporters in sequenced genomes from all domains of life. *Nucleic Acids Res* **2017,** 45, D320-D324.
3. El-Gebali, S.; Mistry, J.; Bateman, A.; Eddy, S.R.; Luciani, A.; Potter, S.C.; Qureshi, M.; Richardson, L.J.; Salazar, G.A.; Smart, A.; Sonnhammer, E.L.L.; Hirsh, L.; Paladin, L.; Piovesan, D.; Tosatto, S.C.E.; Finn, R.D., The Pfam protein families database in 2019. *Nucleic Acids Res* **2019,** 47, D427-D432.
4. Jeong, H.; Barbe, V.; Lee, C.H.; Vallenet, D.; Yu, D.S.; Choi, S.H.; Couloux, A.; Lee, S.W.; Yoon, S.H.; Cattolico, L.; Hur, C.G.; Park, H.S.; Segurens, B.; Kim, S.C.; Oh, T.K.; Lenski, R.E.; Studier, F.W.; Daegelen, P.; Kim, J.F., Genome sequences of Escherichia coli B strains REL606 and BL21(DE3). *J Mol Biol* **2009,** 394, 644-52.
